# Treatment with Oral Active Vitamin D Is Associated with Decreased Risk of Peritonitis and Improved Survival in Patients on Peritoneal Dialysis

**DOI:** 10.1371/journal.pone.0067836

**Published:** 2013-07-02

**Authors:** Julia Kerschbaum, Andreas Vychytil, Karl Lhotta, Friedrich C. Prischl, Martin Wiesholzer, Veronika Machhold-Fabrizii, Gertrude Kopriva-Altfahrt, Christoph Schwarz, Peter Balcke, Rainer Oberbauer, Reinhard Kramar, Paul König, Michael Rudnicki

**Affiliations:** 1 Department of Internal Medicine IV, Nephrology and Hypertension, Medical University Innsbruck, Innsbruck, Austria; 2 Division of Nephrology, Department of Medicine III, Medical University Vienna, Vienna, Austria; 3 Department of Nephrology and Dialysis, Academic Teaching Hospital Feldkirch, Feldkirch, Austria; 4 Clinical Division of Nephrology, Department of Medicine IV, Klinikum Wels-Grieskirchen, Wels, Austria; 5 Division of Nephrology and Dialysis, First Department of Medicine, A.ö. Krankenhaus St. Pölten, St. Pölten, Austria; 6 Division of Nephrology and Dialysis, Department of Medicine VI, Wilhelminenspital, Vienna, Austria; 7 Department of Nephrology, Krankenhaus der Elisabethinen, Linz, Austria; 8 Austrian Dialysis and Transplant Registry, Linz, Austria; Universidade de São Paulo, Brazil

## Abstract

Peritonitis is a major complication of peritoneal dialysis (PD) being associated with hospitalization, catheter loss, technique failure, and increased mortality. Data on incidence rates and risk factors for peritonitis episodes vary between centers. In seven Austrian PD units clinical and laboratory data on each peritonitis episode were collected from all patients (n = 726) who performed PD between January 2000 and December 2009. The peritonitis incidence rate was 0.32 episodes/patient-year. In a multivariate analysis the risk of peritonitis was decreased by 57% in patients treated with oral active vitamin D (HR 0.43; 95% CI 0.28–0.64). Renal disease classified as “other or unknown” (HR 1.65; 95% CI 1.08–2.53) and serum albumin <3500 mg/dl (HR 1.49; 95% CI 1.04–2.15) were also associated with an increased risk of peritonitis. Albumin levels <3500 mg/dl (HR 1.89; 95% CI 1.13–3.17), age (HR 1.06 per year; 95% CI 1.03–1.09), and cardiomyopathy (HR 3.01; 95% CI 1.62–5.59) were associated with increased mortality, whereas treatment with oral active vitamin D was associated with a significantly lower risk of death (HR 0.46; 95% CI 0.27–0.81). In this retrospective multi-center study we identified several factors being related to increased risk of peritonitis in PD patients. Treatment with oral active vitamin D was identified as being independently associated with decreased risk of peritonitis, and decreased all-cause mortality in PD patients.

## Introduction

Peritonitis represents the main acute complication of peritoneal dialysis (PD) and is a leading cause of hospitalization [1], catheter loss and technique failure [2]. It may also lead to death in PD patients [3]. Peritonitis has been described as one of the leading causes of transfer to hemodialysis (HD). The decline of peritonitis rates during the last decades has mostly been achieved by improvements in PD technique such as the introduction of the Y-set-twin-bag connection system (reviewed in [4]). The 2010 Peritonitis Guidelines of the International Society for Peritoneal Dialysis (ISPD) emphasized the importance of measures to be taken to prevent peritonitis episodes [5]. It is recommended to monitor the frequency of PD-associated peritonitis and to carefully analyze each episode, which - on the long term - might help to identify risk factors and improve patients’ outcome.

Several clinical and demographic factors have been reported previously to be associated with an increased risk of peritonitis (reviewed in [6], [7]). Data regarding the association of concomitant medication on the frequency of PD-associated peritonitis and on clinical outcome in PD patients are limited. Although therapy with oral active vitamin D seems to be associated with improved outcome in hemodialysis patients [8], data on PD patients are limited. In a previous study we showed a beneficial association between therapy with oral active vitamin D and decreased rate of peritonitis in a single center cohort of PD patients [9]. Furthermore, immunosuppression was observed to increase the risk of peritonitis in PD patients [10]. No significant effect on the risk of peritonitis could be observed in patients using Sevelamer as a phosphate-binder [11]. The aim of this study was to analyze the peritonitis rates in a large multi-center patient cohort over a 10 year follow-up period. In addition, we aimed to identify risk-factors and concomitant medications, which were associated with increased peritonitis rates and also increased mortality.

## Materials and Methods

### Patients

Medical records of patients treated in seven Austrian PD-units were reviewed for clinical data of patients with end-stage kidney failure who performed PD between January 1, 2000, and December 31, 2009 (five centers) and between January 1, 2006, and December 31, 2009 (two centers). Only centers with more than 10 prevalent PD patients in each year were included in this project. The study population included both prevalent (15.4%) and incident (84.6%) patients. The study was limited to patients over the age of 16 years who (1) were stable on PD for >90 days, (2) did not interrupt PD for >90 days, and were not lost to follow-up until reaching one of the predefined endpoints or the end of the study. Study termination was prespecified as death, kidney transplantation, and transfer to hemodialysis or end of study period. Patients who interrupted PD for more than 90 days and restarted PD-treatment were counted twice (n = 6). All but one center used conventional catheter placement techniques (one center: Moncrief-Popovich technique).

### Ethics Statement

For this study only diagnostic data were used, which were routinely stored in the hospital databases following Austrian law. Therefore, informed consent for storage of personalized data was not needed. These data were anonymized prior to statistic analysis at the Medical University Innsbruck, and these data were collected and used solely for this study. The Institutional Review Board of the Medical University of Innsbruck and the local Institutional Review Boards of the participating centers approved the anonymized analysis of these data; therefore informed consent for the use of personalized information for research purposes was not needed.

### Data Collection

The following baseline-data were collected retrospectively: data on demographic and clinical characteristics such as age, gender, height, weight, blood pressure, primary renal disease, date of start of PD, mode of PD, and comorbidities (diabetes, hypertension, myocardial infarction, heart failure, peripheral vascular disease, stroke, peripheral arterial disease). Furthermore, we collected baseline-laboratory parameters such as serum albumin, hemoglobin, calcium, phosphate, parathyroid hormone (PTH). We extracted the use of concomitant medication such as renin-angiotensin-aldosterone-system (RAAS) inhibitors, oral active vitamin D (i.e. calcitriol and alfacaldidol), phosphate binders and statins before the first episode of peritonitis from the original patient records. Any patient that had received one type of comedication prior to the first episode of peritonitis was retained in that co-medication cohort for all further analyses (i.e. in an intention-to-treat manner; therefore dosage of oral active vitamin D was not obtained). The diagnosis of peritonitis was established in all centers based on two or more of the following findings: (1) abdominal pain, (2) cloudy effluent, (3) effluent white blood cell count exceeding 100/µL with at least 50% neutrophils, (4) positive Gram staining. Number and date of peritonitis episodes, and culture results were obtained in order to calculate peritonitis incidence rates. Clinical follow-up included transplantation, switch to hemodialysis, continued PD, or death. The date of all-cause death was extracted from the Austrian Dialysis and Transplant Registry ^31^. Peritoneal dialysis patients were treated for secondary hyperparathyroidism with oral vitamin D analogues (referred to as “oral active vitamin D”) according to local policies. Most centers used a (modified) version of the clinical practice guidelines from the Kidney Disease Outcomes Quality Initiative (K/DOQI: serum level of intact PTH >300 pg/mL, serum phosphorus <1.78 mmol/L, serum calcium <2.37 mmol/L).

### Statistical Analysis

Peritonitis incidence rate was calculated using all episodes of peritonitis during the study period and the total amount of patient years during follow-up. The frequency of PD-associated peritonitis in each year was calculated as median of episodes per patient–year (and interquartile range). The distribution of microorganisms causing peritonitis episodes was calculated on the basis of the total number of identified organisms.

For identifying risk factors for peritonitis, only the first episode of peritonitis was regarded. Baseline characteristics are given as median and range, and as frequency, respectively. The two-tailed Mann-Whitney U-test with a confidence interval (CI) of 95% was used for analysis of significance between metric variables, and the two-tailed chi-square test was used for investigating associations between categorical variables. A univariate Cox proportional hazards model was used to determine independent risk factors for peritonitis and all-cause death. Multivariate analysis included all identified risk factors in univariate analysis, center and year of start of PD. All laboratory data were categorized into three categories according to the K/DOQI guidelines (KDOQI). As non-linear effects were expected, laboratory data as well as primary renal disease, and the mode of dialysis (CAPD, APD, switch from one to another) were introduced as indicator variables. To minimize bias due to missing data, multiple imputation was used to estimate missing values of parathyroid hormone and BMI. In addition, propensity scores were calculated to exclude bias by differential assignment to treatment with oral active vitamin D. The analysis was performed using SPSS for Windows (version 18.0; SPSS, Cambridge, MA, U.S.A.).

## Results

### Peritonitis Incidence Rate and Culture Results

We collected data from 726 patients (6 patients who discontinued PD for a period >90 days were counted twice, i.e. n = 720 individual patients), representing 1703 patient years and 44.3% of the PD population treated in Austria in this period. Table 1 shows the baseline characteristics of the whole cohort, and of patients who did or did not experience at least one episode of peritonitis. Median age of patients was 55.4 (16.1–87.0) years and 58.8% were males. Overall, 44.8% of patients experienced no episode of peritonitis and 55.1% of patients had at least one episode of peritonitis. Five hundred fifty-two episodes of peritonitis were diagnosed, resulting in a peritonitis incidence rate of 0.32 episodes/patient-year. Median rates of peritonitis stratified by year did not differ significantly between the evaluated years. Of 602 pathogens isolated in 552 episodes of peritonitis, 335 (55.7%) were gram-positive and 123 (20.4%) were gram-negative. In 20 cultures (3.3%) Candida species were isolated, and 97 cultures showed negative results (16.1%). In 4.0% of cases the underlying germ was reported as unknown (table 2).

**Table 1:** Patient characteristics at baseline.

Characteristic	Whole cohort	Without Peritonitis	With Peritonitis	p-value
*Clinical data*				
Total (n)	726	326	400	–
Male gender (726)	58.8%	62.8%	54.0%	0.017
BMI (kg/m^2^)^a^ (291)	24.0 (12.5−39.1)	24.1 (16.4−38.8)	23.9 (12.5−39.1)	0.944
Age (years)^a^ (726)	55.7 (16.1−87.0)	55.2 (16.1−87.0)	56.2 (16.5−83.4)	0.550
Primary renal disease (726)				0.047
GN	23.9%	27.3%	19.6%	
DN	16.1%	13.8%	19.0%	
PCKD	9.4%	10.0%	9.2%	
Other (e.g. interstitial nephritis)/unknown	50.6%	49.0%	52.1%	
Modality (725)				0.282
CAPD only	60.1%	57.9%	62.6%	
APD only	27.3%	29.8%	24.5%	
Switch^b^	12.6%	12.3%	12.9%	
*Comedication*				
RAAS inhibitors (718)	63.5%	64.7%	62.0%	0.457
Statins (714)	43.7%	43.7%	43.8%	0.980
Immunosuppression (715)	16.9%	17.5%	16.2%	0.641
Oral active Vitamin D (716)	69.0%	69.0%	68.9%	0.979
Calcium-based PB (716)	52.0%	48.7%	56.5%	0.038
Non calcium-based PB (716)	40.3%	39.3%	41.3%	0.594
*Laboratory data*				
Albumin (447)				0.104
<3500 mg/dl	35.3%	32.1%	39.6%	
3500–5200 mg/dl	64.7%	67.8%	60.4%	
Calcium (614)				0.787
<2.10 mmol/l	21.3%	21.2%	21.1%	
2.10–2.37 mmol/l	49.9%	51.0%	48.7%	
>2.37 mmol/l	28.8%	27.7%	30.2%	
Phosphate (617)				0.430
<1.13 mmol/l	8.9%	9.1%	8.7%	
1.13–1.78 mmol/l	39.2%	41.3%	36.6%	
>1.78 mmol/l	51.9%	49.6%	54.7%	
Parathyroid hormone (214)				0.501
<150 pg/ml	32.6%	34.5%	30.5%	
150–300 pg/ml	33.5%	35.3%	31.6%	
>300 pg/ml	34.0%	30.3%	37.9%	
Hemoglobin (622)				0.147
<10 g/dl	12.4%	11.5%	13.5%	
10–12 g/dl	58.8%	56.6%	61.7%	
>12 g/dl	28.8%	31.9%	24.8%	
*Comorbid conditions*				
Diabetes (719)	24.1%	23.0%	25.4%	0.453
Hypertension (719)	82.2%	81.6%	83.0%	0.624
Cardiomyopathy (719)	13.1%	14.6%	11.1%	0.166
Myocardial infarction (719)	10.2%	11.4%	9.0%	0.295
Cerebrovascular disease (719)	9.6%	10.1%	9.0%	0.611
Peripheral arterial disease (719)	13.2%	11.4%	15.8%	0.083

a median and range.

b switch from CAPD to APD or vice versa.

DN; diabetic nephropathy. GN; glomerulonephritis. PB; phosphate binders. PCKD; polycystic kidney disease. A p-value <0.05 was considered as significant. Numbers in brackets in the first column represent the number of patients for who data were available.

**Table 2:** Distribution of identified microorganisms.

	N (total = 602)	% of total
**Gram-positive bacteria**	**335**	**55.7**
CNS	139	23.1
Staph. aureus	87	14.5
Streptococcus	57	9.5
Enterococcus	23	3.8
Corynebacterium	14	2.3
Other	15	2.5
**Gram-negative bacteria**	**123**	**20.4**
E. coli	36	6.0
Pseudomonas	14	2.3
Enterobacter	8	1.3
Other	65	10.8
**Candida species**	**20**	**3.3**
**Culture negative**	**97**	**16.1**
Unknown	24	4.0

CNS; coagulase-negative staphylococcus. E. coli; Escherichia coli. Staph. aureus; Staphylococcus aureus. Sum does not add up to 100% due to rounding.

### Analysis of Risk Factors for Peritonitis

**Figure 1:**
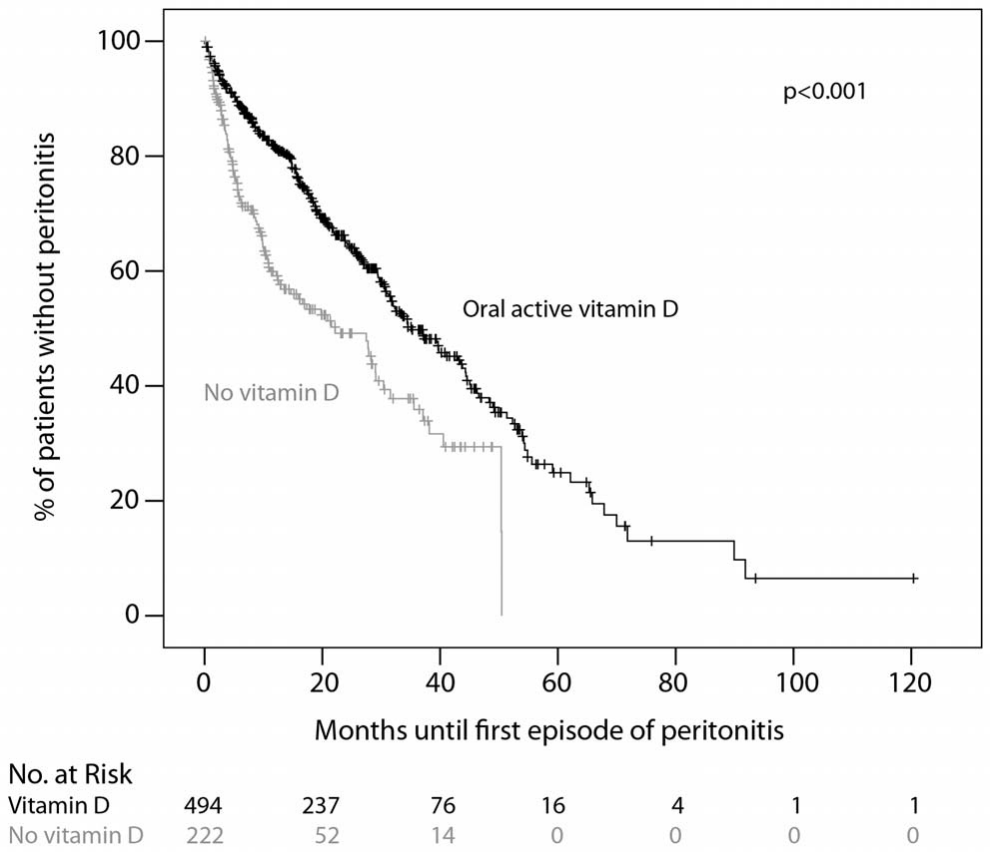
Kaplan-Meier analysis for time to first episode of peritonitis. Patients treated with oral active vitamin D at start of PD (black, n = 494) were compared with untreated patients (grey, n = 222). Data on vitamin D treatment were available for n = 716 patients.

In a univariate Cox proportional hazards model the following demographic, clinical and laboratory parameters were associated with an increased risk for peritonitis: Albumin levels below 3500 mg/dl, higher age, underlying renal disease (diabetic nephropathy), peripheral arterial disease, diabetes as a comorbid condition. Hemoglobin levels >12 g/dl were associated with a decreased risk for of peritonitis. Regarding concomitant medication, only treatment with oral active vitamin D was associated with a significantly lower risk of peritonitis (HR 0.55; 95% CI 0.43–0.70). As shown in figure 1 the time to the first episode of peritonitis was significantly longer in patients treated with oral active vitamin D (median: 16.3 months vs. 5.5 months).

We then used a multivariate Cox regression hazards model including all significant factors from univariate analysis (table 3). Center and year of start of PD were also included as covariates to minimize a center- or an era-effect. The risk of peritonitis was decreased by 57% in patients treated with oral active vitamin D (HR 0.43; 95% CI 0.28–0.64; adjusted for albumin, hemoglobin, age, renal disease, peripheral vascular disease, diabetes, center, and year of start of PD). In addition, patients with renal diagnosis classified as “other (e.g. interstitial nephritis)” or “unknown” had a 1.65-fold increased risk of peritonitis (HR 1.65; 95% CI 1.08–2.53). Furthermore, albumin levels <3500 mg/dl were also associated with an increased risk of peritonitis (HR 1.49; 95% CI 1.04–2.15).

**Table 3:** Multivariate analysis of risk factors for peritonitis.

Characteristic	HR	95% CI	p-value
Oral active Vitamin D	0.43	0.28–0.64	<0.001
Primary renal disease other/unknown	1.65	1.08–2.53	0.030
Albumin <3500 mg/dl	1.49	1.04–2.15	0.012

Detailed analysis revealed that oral active vitamin D especially reduced the risk for gram-positive (HR 0.51; 95% CI 0.36–0.72) and gram-negative (HR 0.44; 95% CI 0.26–0.73) episodes of peritonitis, whereas in culture-negative peritonitis episodes, no significant association was seen (HR 0.84; 95% CI 0.46–1.52). Propensity scores for the probability of differential assignment to treatment with oral active vitamin D were calculated. Repeated analysis including propensity scores did not change the results (data not shown). In patients treated with oral active vitamin D peritonitis rate was 0.29 episodes/patient-year, whereas in patients without vitamin D the rate was 0.45 episodes/patient-year.

### Analysis of Risk Factors for All-cause Death

**Figure 2:**
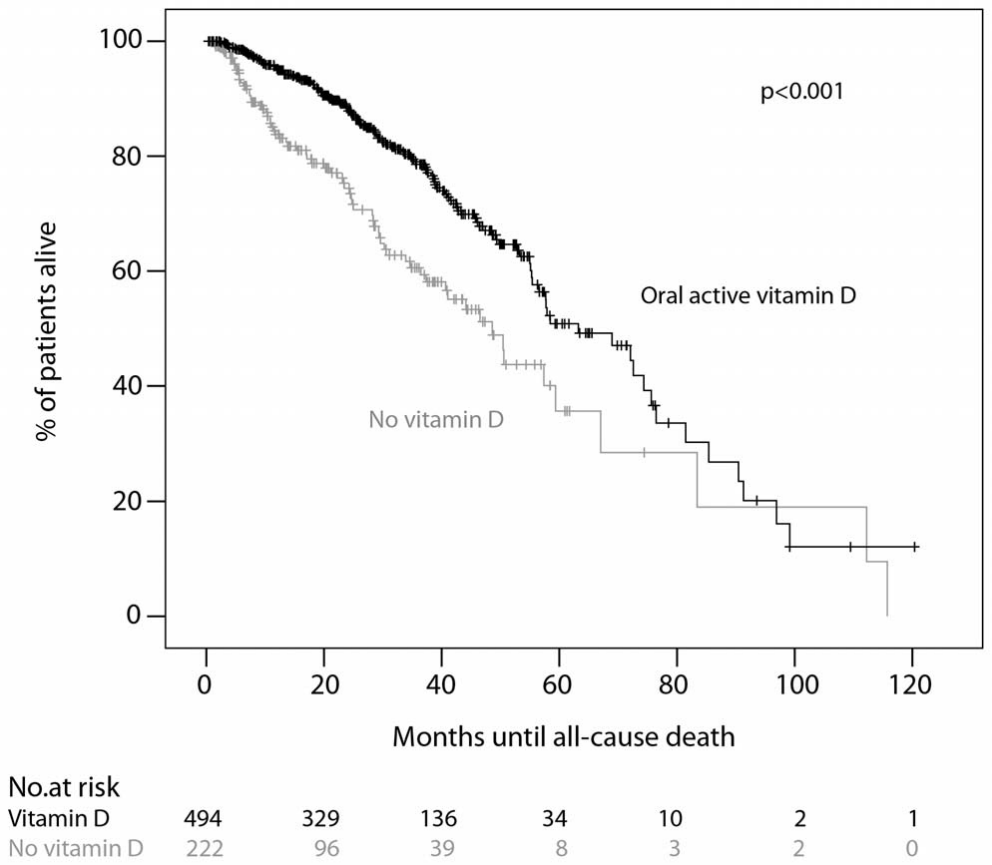
Kaplan-Meier analysis of survival. Survival curves of patients treated with oral active vitamin D at start of PD (black, n = 494) were compared with untreated patients (grey, n = 222). Data on vitamin D treatment were available for n = 716 patients.

The overall mortality rate was 11.4%/year (17.4% in the subgroup of patients without vitamin D, and 9.6% in the group of patients with oral active vitamin D treatment, respectively). In a univariate Cox proportional hazards model for the risk of all-cause death an increase in age per year and underlying renal disease (diabetic nephropathy and other/unknown) as well as albumin levels below 3500 mg/dl were significantly associated with increased mortality, whereas calcium levels >2.37 mmol/l and female gender were significantly associated with decreased risk of death. Patients with peripheral arterial disease, diabetes as a comorbid condition, cardiomyopathy, previous myocardial infarction or cerebrovascular disease had a significantly increased risk for all-cause mortality. With regard to concomitant medication, treatment with oral active vitamin D was associated with a significantly lower risk of death (HR 0.54; 95% CI 0.40–0.72) as well as treatment with non calcium-based phosphate binders (HR 0.59; 95% CI 0.43–0.80). Figure 2 shows the time to death according to the use of oral active Vitamin D. Neither peritonitis itself nor the group of identified microorganisms (gram-positive, gram-negative, sterile) were significantly associated with the risk for all-cause death (adjusted for identified risk factors for death; data not shown).

We then used a multivariate Cox regression hazards model including all significant factors identified in univariate analysis to evaluate independent risk factors for all-cause mortality (table 4). As in the former analysis we also included center and year of start of PD as covariates. Mortality was decreased by 54% in patients treated with oral active vitamin D (HR 0.46; 95% CI 0.27–0.81; adjusted for age, gender, underlying renal disease, albumin, calcium, peripheral arterial disease (PAD), diabetes, cardiomyopathy, previous myocardial infarction, cerebrovascular disease, the use of non calcium-based phosphate binders, center, and year of start of PD). In addition, albumin levels <3500 mg/dl (HR 1.89; 95% CI 1.13–3.17), increasing age (HR 1.06 per year; 95% CI 1.03–1.09), and cardiomyopathy (HR 3.01; 95% CI 1.62–5.59) were associated with an increased risk for all-cause mortality. In summary, oral active vitamin D reduced the risk of peritonitis (especially of gram-positive and gram-negative peritonitis) and the risk for all-cause mortality in PD-patients significantly (table 5).

**Table 4:** Multivariate analysis of risk factors for all-cause death.

Characteristic	HR	95% CI	p-value
Oral active Vitamin D	0.46	0.27–0.81	<0.001
Age (per year increase)	1.06	1.03–1.09	0.004
Cardiomyopathy	3.01	1.62–5.59	0.017
Albumin <3500 mg/dl	1.89	1.13–3.17	0.002

**Table 5:** Vitamin D and the risk for peritonitis or all-cause death.

	No vitamin D	Oral active vitamin D
Risk for the 1^st^ episode of peritonitis	Ref	0.43 (95% CI 0.28–0.64)
Risk for all–cause death	Ref	0.46 (95% CI 0.27–0.81)
Risk for gram-positive peritonitis	Ref	0.51 (95% CI 0.36–0.72)
Risk for gram-negative peritonitis	Ref	0.44 (95% CI 0.26–0.73)
Risk for sterile peritonitis	Ref	0.84 (95% CI 0.46–1.52)
Time to 1^st^ episode of peritonitis (months)^a^	5.5 (2.8–30.6)	16.3 (6.3–30.6)^b^
Episodes of peritonitis/patient-year	0.45	0.29

a median (interquartile range);

b P<0.001.

## Discussion

Our study shows for the first time that treatment with oral active vitamin D might reduce the risk of peritonitis and the risk of all-cause death in PD-patients, which was shown in this large multi-center cohort study. In the past few years the effects of treatment with vitamin D and particularly of 1,25-dihydroxy-cholecalciferol on the cardiovascular system and on the mortality of patients with end-stage renal disease have been discussed controversially. On the one hand concerns have been raised that treatment with high doses of vitamin D is associated with an increased risk of vascular calcifications [12]–[14]. On the other hand studies suggest that treatment with vitamin D and vitamin D analogues might be associated with substantial health benefits in dialysis patients. In a historical cohort study including 51,037 chronic hemodialysis patients, those subjects who received injectable vitamin D had a 2-year survival benefit of 20% (HR 0.80; 95% CI 0.76–0.83) [15]. Shoji et al. reported a significantly reduced risk of cardiovascular death in a small (n = 242) cohort of hemodialysis patients who received treatment with a median dose of 0.5 µg oral 1-alphacalcidol over a follow-up of 61+/−23 months (HR 0.287, 95% CI 0.127–0.649), while death from non-cardiovascular causes was not different between both groups [16]. Also therapy with oral active vitamin D has been reported to be associated with improved survival benefits in hemodialysis patients. In a large multicenter retrospective registry study in six Latin America countries, treatment with oral calcitriol (n = 7203) as compared to no treatment (n = 8801) was associated with a lower risk of all-cause death (HR 0.55, 95% CI 0.49–0.63) [8].

Beside its role in the calcium and phosphate homeostasis, calcitriol exhibits numerous pleiotropic effects in various organ systems. Since more than 30 cell types express the vitamin D receptor (VDR), and more than 10 organs are capable of paracrine 1-α-hydroxylation, it is not surprising that vitamin D status or therapy has been linked to the renin-angiotensin-aldosterone system, inflammation, auto- and innate immunity, and neoplastic diseases, respectively (reviewed in [17]). Uremia represents a state of chronic inflammation, and evidence exists that vitamin D influences lymphocyte and macrophage activities, and also cytokine release. The activation of the intracellular antimicrobial peptide cathelicidin in monocytes and macrophages by toll-like receptors is vitamin D3-dependent [18], which leads to enhanced killing of intracellular *Mycobacterium tuberculosis* [19]. On the other hand, vitamin D has been shown also to have immunosuppressive properties. Cohen *et al.* demonstrated a potent inhibitory effect of active vitamin D3 on tumor necrosis factor α-stimulated macrophages from PD patients [20]. Based on these observations one may speculate that a repleted vitamin D status or treatment with active vitamin D may represent one of many requirements for the amelioration of a pathologically over-reactive or a suppressed immune system. However, one has to mention that in a retrospective study only associations can be described, which do not necessarily reflect causality. Although existing evidence supports the idea of beneficial effects of vitamin D in various patient cohorts, our study is the only one, which evaluated the effect of oral active vitamin D in a large cohort of PD patients. Before more substantial conclusions are drawn, these results need confirmation in further studies.

Beside the novel finding regarding the association of treatment with oral active vitamin D with decreased risk of peritonitis and all-cause mortality, low serum albumin levels (<3.5 g/dl) were associated with an increased risk of peritonitis and increased risk of death, as was the diagnosis of cardiomyopathy. Low levels of serum albumin have been shown to be associated with the risk of peritonitis in several previous studies [21], [22]. It might be hypothesized that hypoalbuminemia as a result of malnutrition, inflammatory response or of uremia itself, may lead to a higher susceptibility to infections. Serum calcium levels significantly differed between patients without and with vitamin D (table 6) and were significantly correlated with levels of albumin. In a multivariate analysis using Backwards Wald Method, only albumin levels but not calcium levels were significantly associated with peritonitis and all-cause mortality. Despite differences in local policies regarding exit-site care in Austrian peritoneal dialysis centers, which were evaluated in a nationwide survey in 2006 [23], the effect of vitamin D described in our manuscript was seen in all participating centers (data not shown).

**Table 6:** Patient characteristics stratified by treatment with active vitamin D.

Characteristic	All patients	No vitamin D	Vitamin D	p-value
*Clinical data*				
Total (n)	726	222	494	–
Male gender (726)	58.8%	61.7%	41.7%	0.390
BMI (kg/m^2^)^a^ (291)	24.0 (12.5–39.1)	23.8 (12.5–36.5)	24.0 (16.4–39.1)	0.556
Age (years)^a^ (726)	55.7 (16.1–87.0)	58.8 (16.1–87.0)	54.6 (16.5–84.9)	0.001
Primary renal disease (726)				0.349
GN	23.9%	25.2%	23.7%	
DN	16.1%	16.7%	15.8%	
PCKD	9.4%	7.2%	10.5%	
Other (e.g. interstitial nephritis)/unknown	50.6%	50.9%	50.0%	
Modality (725)				0.399
CAPD only	60.1%	62.0%	59.1%	
APD only	27.3%	28.1%	27.3%	
Switch^b^	12.6%	10.0%	13.6%	
*Comedication*				
RAAS inhibitors (718)	63.5%	54.5%	67.6%	0.001
Statins (714)	43.7%	40.0%	45.6%	0.139
Immunosuppression (715)	16.9%	17.6%	16.6%	0.758
Calcium-based PB (716)	52.0%	49.5%	53.4%	0.335
Non calcium-based PB (716)	40.3%	26.6%	46.4%	<0.001
*Laboratory data*				
Albumin (447)				<0.001
<3500 mg/dl	35.3%	55.6%	28.6%	
3500–5200 mg/dl	64.7%	44.4%	71.4%	
Calcium (614)				0.015
<2.10 mmol/l	21.3%	28.7%	18.2%	
2.10–2.37 mmol/l	49.9%	44.8%	52.1%	
>2.37 mmol/l	28.8%	26.5%	29.7%	
Phosphate (617)				0.218
<1.13 mmol/l	8.9%	12.1%	7.7%	
1.13–1.78 mmol/l	39.2%	37.9%	39.8%	
>1.78 mmol/l	51.9%	50.0%	52.6%	
Parathyroid hormone (214)				0.031
<150 pg/ml	32.6%	40.6%	28.5%	
150–300 pg/ml	33.5%	37.9%	31.9%	
>300 pg/ml	34.0%	21.7%	40.0%	
Hemoglobin (622)				0.071
<10 g/dl	12.4%	10.8%	12.9%	
10–12 g/dl	58.8%	65.9%	56.1%	
>12 g/dl	28.8%	23.2%	30.9%	
*Comorbid conditions*				
Diabetes (719)	24.1%	25.9%	23.1%	0.421
Hypertension (719)	82.2%	80.0%	83.0%	0.341
Cardiomyopathy (719)	13.1%	20.9%	9.7%	<0.001
Myocardial infarction (719)	10.2%	11.8%	9.5%	0.353
Cerebrovascular disease (719)	9.6%	12.3%	8.5%	0.117
Peripheral arterial disease (719)	13.2%	18.2%	11.0%	0.008

a median and range.

b switch from CAPD to APD or vice versa. DN; diabetic nephropathy. GN; glomerulonephritis. PB; phosphate binders. PCKD; polycystic kidney disease. A p-value <0.05 was considered as significant. Numbers in brackets in the first column represent the number of patients for who data were available.

Despite these interesting and encouraging results this study has several limitations. As a retrospective study these results are subject to bias by indication, i.e. serum calcium, phosphorus, and PTH levels were significantly different between patients who received treatment with oral active vitamin D and who did not. However, these characteristics demonstrate adequate treatment according to the K/DOQI-guidelines, since patients with moderately high levels of serum calcium and phosphorus and with no important serum PTH elevations did not receive oral active vitamin D treatment. Although every effort has been made in the multivariate analysis to adjust for confounders such as age, comorbidities, other comedication and laboratory values, we cannot ultimately rule out the possibility that to "healthier" patient characteristics in the vitamin D cohort contributed to improved survival of these patients. Furthermore, we did not have data on serum-albumin from a considerable amount (38%) of the patients. We did not collect data on markers of bone turnover, vascular calcification, inflammatory status, and we did not analyze the effect of socioeconomic status, which could represent confounders in this analysis. Furthermore, therapy with oral active vitamin D was only recorded at the begin of PD treatment, and therefore we cannot provide data about the cumulative dosage. The levels of serum 25-hydroxyvitamin D were not analyzed due to a high number of missing values. Propensity scores for the probability of differential assignment to treatment with oral active vitamin D were calculated and did not change the results (data not shown).

## Conclusion

In this large multi-center retrospective cohort study we identified several factors being related to increased risk for peritonitis in PD patients. Oral active vitamin D was independently associated with decreased risk for peritonitis and all-cause mortality in PD patients. This effect appeared to be independent of other potential risk factors and confounders. Despite these promising findings, further studies will have to be done to validate these results.
